# Lecture on the Teeth

**Published:** 1921-03

**Authors:** Alfred A. Crocker


### Lecture on the Teeth

By ALFRED A. CROCKER

AS we all understand, the teeth are coming in more and more for their share of attention, and what used to be disregarded in their care is now regarded as most important. The reason for this is that we now know positively that the teeth have a great deal to do with our health and general well being. Instead of thinking, "Oh, well, if I lose that tooth I can get a false one which will do just as well," we now take notice and say, "I can save that tooth if I consult a dentist." False teeth are nothing more or less than makeshifts to take the place of lost teeth, just the same as false legs and arms or false eyes are. But we seem to be alert to take much better care of our arms, legs and eyes than of our teeth. Possibly it is because we think we can get by in fooling most of the people with decayed teeth or false ones and not realize that it all can be prevented just as easily as we prevent loss of our arms and legs. Possibly it is because the subject of saving the teeth by preventive dentistry has not been brought up in the right way, or because the need of seeing a dentist in consultation on tooth conditions has not been considered in an equal degree as the need of calling in a physician when you feel ill. This is natural, however, and is because tooth decay, which is one of the causes of ultimate loss of the teeth, is painless, and the process of decay, if not prevented or stopped, continues until the tooth aches, and then dental surgical treatment is the only means left of saving the utility of the tooth.

Thus we go on thinking we are all right until suddenly a terrific pain starts up—then to the dentist we go. From a dentist's point of view this is most lamentable, although we have been doing what we thought was all right; but in reality it was just the wrong thing.

Now the prevention of decay is so important that the dental profession recently recognized the great work of Dr. W. D. Miller, by placing a monument to his memory on the grounds of the Ohio State University. Dr. Miller did a great deal of research work on dental subjects and especially on prevention of decay, and brought the subject up in such a form that when one once grasps the idea of preventing decay, one can actually do it. This requires periodical examination of the teeth by a dentist at least twice a year, so as to stop the advance of decay if any has started. Home care of the teeth by use of tooth brush and dentifrices is commendable and necessary for cleanliness, but absolutely useless so far as preventing decay, as most of us have found out; for decay takes place in spite of all household attempts to prevent it. Why is this? Simply that the decay starts in the crevices between the teeth, where the home efforts to keep them clean do not reach. For this reason it becomes necessary to see a dentist regularly for tooth cleaning and examination and detection of decay, if any.

A dentist can properly examine the teeth, where it is impossible for the individual to do it himself.

Decay after it gets started, and not interfered with or stopped by the dentist by a filling, continues on its course of destruction and eventually finishes by the tooth reaching the nerve pulp—and a terrific pain announces this stage. If one still refuses to go to a dentist the tooth is doomed, and very often it is too late and the only way to save the utility of the tooth is to save the tooth structure by extracting the nerve and thus leaving a dead tooth in the mouth. This is often done, and by the present method of root canal filling and treatment the tooth lasts very well for years, especially if the patient is young. However, after middle life the ultimate abscess of this tooth begins to accrue. This is where the periodical visit to a dentist again comes in very valuable for the patient, because a dentist looks at this feature of teeth with root-canal filling, and at periods X-rays them to detect the oncoming of the abscess. You may ask why that abscess comes. All we know about it is that it is nature's method of getting rid of a dead tooth. Then you may ask, why did not the dentist extract it at first? According to present-day ideas, the opinion is divided, but the consensus of opinion is that it is good dentistry to save the dead tooth for its utility as long as possible, for as long as it is in healthful condition it is far better than a false tooth, both in utility and beauty of appearance. People who have followed the preventive dentistry method have very few fillings in their teeth and those are small, and besides they generally have their own teeth. People who have paid some attention, but not a great deal, to their teeth have large fillings and some false teeth, and perhaps later on will have all false teeth. People who disregard all attention to their teeth have a hard time of it when at last they have to go to a dentist.

The accepted theory of decay is that it is the result of localized acid conditions in the mouth, caused by fermentative action of small bacteria held on the surface of the enamel of the teeth by filmy plaques of food substances. Between the teeth, where the tooth brush or floss have missed, these plaques harbor the acid, forming conditions which dissolve out the lime salts of the enamel and the dentine or tooth structure, making additional lodgment space in the cavity as it proceeds for additional bacteria. When it is understood and realized that this germ of caries or decay multiplies at the rate of four million per minute, you will realize what an active enemy you have to deal with in preventing decay. The idea of going to a dentist twice a year is to permit him to detect the places where decay has started and to fill the small cavities. It takes about a year of continual activity of the decaying process to reach the pulp of the tooth after starting at the enamel of the tooth, so you see an examination every six months leaves a good margin of safety for the vitality of the tooth.

To show you the value of regular inspection of the teeth for detection of decay, and general cleaning to prevent further decay, stopping what cavities there were with fillings, I ask you to study the following table:

| | 1907 1908 | 1908 1909 | 1909 Nov. 1910 | Nov. 1910 Apr. 1911 | Apr. 1911 Apr. 1912 | Apr. 1912 Apr. 1913 | Apr. 1913 Apr. 1914 | Apr. 1914 Jan. 1915 |
|---|---|---|---|---|---|---|---|---|
| Diphtheria | 6 | 2 | 1 | 0 | 0 | 0 | 1 | 0 |
| Mumps | 8 | 3 | 10 | 4 | 0 | 0 | 0 | 0 |
| Scarlet Fever | 17 | 8 | 12 | 8 | 0 | 0 | 0 | 0 |
| Pneumonia | 3 | 5 | 4 | 6 | 0 | 0 | 0 | 0 |
| Measles | 24 | 50 | 40 | 25 | 0 | 0 | 6 | 0 |
| Tonsilitis | 19 | 16 | 8 | 3 | 0 | 0 | 0 | 0 |
| Whooping Cough | 7 | 2 | 2 | 0 | 0 | 0 | 0 | 0 |
| Chicken Pox | 15 | 17 | 10 | 6 | 0 | 0 | 0 | 0 |
| Croup | 4 | 0 | 0 | 0 | 0 | 0 | 0 | 0 |

This is the record kept of children's diseases in an orphan asylum before and after work and periodical prophylaxis of the teeth was started. Work was started in November, 1910, and in April, 1911, the mouth of the last child had been put in order. Notice that for nearly four years after the starting of dental care the diseases of children had been practically eliminated. The six cases of measles occurring during 1913 were caused by a new child, whose mouth was in bad condition on entering; and the children inspected were all in need of additional dental work. The one case of diphtheria was also from a new child with bad teeth.

Similar results are found after dental care of adults. These remarkable results are also reiterated in the report from a large industry, where 2,002 men and women were employed. Dental work was started in 1917. Out of the 2,002 patients it was found that there were 9,139 cavities, or an average of 4.6 cavities per patient. There were 1,230 badly decayed teeth, or an average of 0.6 per patient. They had lost 9,320 teeth, or an average of 4.7 per individual. Of those examined 951, or 47.5 per cent., showed inflamed gums, 68 had abscessed teeth, with pus flowing into the mouth, and 161 additional cases showed pyorrhea, of which 62 had reached the pus stage. These figures are quoted not because they are in any way indicative of an excessive amount of dental impairment, but rather as an indication of what may be expected in any representative group of similar size. The first work attended to in all these patients was to place all teeth in good condition by filling all cavities, treating all abscesses and pyorrhea condition, and having false teeth to replace the vacant spaces. After this work was done by each patient's family dentist, the institute dentist who had diagnosed each case and recommended the work that had been done, checked up each case; and thereafter by the periodical prophylaxis and detection of additional decay, if any started, kept all these patients in good mouth condition and materially helped their health and increased their production. This work showed the first year reduction in the following ailments of remarkable percentage: Neuralgia and Neuritis 75 per cent., Colds 16 per cent., Headaches 52 per cent., Skin Troubles 53 per cent., Nervous Troubles 40 per cent.

The next subject for our consideration, one which if not watched and prevented also leads to the loss of the teeth is pyorrhea. Cleanliness of the teeth is absolutely necessary to prevent it, that is, the pathogenic germs found normally in the mouth should be continually under control. Deposits of tartar on the teeth are a frequent cause of incipient pyorrhea, and if not removed and the gums healed lead to the further stages of the trouble. Of course, where this progresses it is because of the neglect by the patient or lack of information by not going to a dentist. While the teeth themselves may be perfect as to fillings and decay, their contact with the gums and jaw bone or alveolar process in which they rest becomes insecure, and the constantly increasing army of bacteria which the white corpuscles of the blood can not control consumes the peridental membrane, which is the safety lining of the tooth root and tooth socket. This gone, necrosis of the bone sets in, and soon the teeth are so loose that the gum alone can't hold them and they drop out. Pyorrhea is caused by the irritation of margins of the gum by tartar, tooth picks, hard tooth brushes, impacted decomposed food particles not promptly removed, injury to the gum from molocclusion. There are other causes, but the ones mentioned are all within the jurisdiction of each patient to prevent.

Another subject I wish to touch upon is the relation of tooth and gum condition to the general health. In discussing decay I mentioned the abscess which eventually comes at the roots of devitalized teeth, and under the subject of pyorrhea the abscessed condition of the peridental membrane and gum sockets. The white corpuscles of the blood in coagulating at these abscessed centers in order to counteract the trouble are killed and accumulate, forming pus which then is a poison and is reabsorbed in the blood stream along with the excess of bacterial production. This goes direct to the heart and thence to all parts of the body. While infection of this kind can take place from any part of the body such infection coming from the teeth and recognized as such has cleared up many cases of neuralgia, neuritis, rheumatism, deafness, insanity, coryza, headache—almost the entire category of bacterial diseases formerly called incurable or chronic.

Another feature of the necessity of care of the teeth is that their original function is to properly prepare the food for digestion and assimilation. A uniform ration of food as required by the body requires a uniform predigestive apparatus and indicates the necessary upkeep of the teeth to maintain sufficient teeth in good order to derive sufficient nourishment from the ration for active participation in your vocation.

The object of this work is to develop an informed public. We more readily accept things that we understand.

